# MGAT3-mediated glycosylation of tetraspanin CD82 at asparagine 157 suppresses ovarian cancer metastasis by inhibiting the integrin signaling pathway

**DOI:** 10.7150/thno.43865

**Published:** 2020-05-16

**Authors:** Jun Li, Jiawen Xu, Luhan Li, Alessandro Ianni, Poonam Kumari, Shuo Liu, Peiqing Sun, Thomas Braun, Xiaoyue Tan, Rong Xiang, Shijing Yue

**Affiliations:** 1 State Key Laboratory of Medicinal Chemical Biology, School of Medicine, Nankai University, Tianjin, China; 2 Department of Cardiac Development and Remodeling, Max-Planck-Institute for Heart and Lung Research, Bad Nauheim, Germany; 3 Department of Cancer Biology, Wake Forest University School of Medicine, Winston-Salem, NC, USA

**Keywords:** Ovarian cancer, Metastasis, Glycosylation, CD82, Exosomes

## Abstract

**Background**: Tetraspanins constitute a family of transmembrane spanning proteins that function mainly by organizing the plasma membrane into micro-domains. CD82, a member of tetraspanins, is a potent inhibitor of cancer metastasis in numerous malignancies. CD82 is a highly glycosylated protein, however, it is still unknown whether and how this post-translational modification affects CD82 function and cancer metastasis.

**Methods**: The glycosylation of CD82 profiles are checked in the paired human ovarian primary and metastatic cancer tissues. The functional studies on the various glycosylation sites of CD82 are performed in vitro and in vivo.

**Results**: We demonstrate that CD82 glycosylation at Asn157 is necessary for CD82-mediated inhibition of ovarian cancer cells migration and metastasis *in vitro* and *in vivo*. Mechanistically, we discover that CD82 glycosylation is pivotal to disrupt integrin α5β1-mediated cellular adhesion to the abundant extracellular matrix protein fibronectin. Thereby the glycosylated CD82 inhibits the integrin signaling pathway responsible for the induction of the cytoskeleton rearrangements required for cellular migration. Furthermore, we reveal that the glycosyltransferase MGAT3 is responsible for CD82 glycosylation in ovarian cancer cells. Metastatic ovarian cancers express reduced levels of MGAT3 which in turn may result in impaired CD82 glycosylation.

**Conclusions**: Our work implicates a pathway for ovarian cancers metastasis regulation via MGAT3 mediated glycosylation of tetraspanin CD82 at asparagine 157.

## Introduction

Ovarian cancer is a frequent gynecological malignancy characterized by a high rate of metastasis and lethality. Differently from other kind of tumors, ovarian cancer only rarely metastasizes through the vasculature, but it rather invades the adjacent tissues or spreads to the distal organs through passive peritoneal dissemination and it is often associated with ascites formation 1-4. The members of integrin family of transmembrane cellular receptors mediate cellular adhesion to the extracellular matrix (ECM) and play crucial roles in the transmission of intracellular signals required for cellular migration.

Cell migration is orchestrated by a highly elaborate complex of molecular pathways, which promotes rapid turnover of the focal adhesion proteins and complex dynamic changes in the cytoskeleton structure, such as the formation of stress fibers and assembly of other specialized structures. Binding of integrins to ECM induces integrin clustering, which in turn leads to the recruitment of cytosolic associated proteins such as the focal adhesion kinase (FAK). Upon recruitment and subsequent autophosphorylation, FAK promotes the phosphorylation of downstream targets, which control the signaling cascade involved in cell migration 5-7. Therefore, integrin-mediated signaling plays an important role in the initiation and establishment of metastasis.

Within the last decade, tetraspanins have been recognized to interfere with cancer metastasis without affecting the growth of the primary tumor. Different studies demonstrated that tetraspanins can directly or indirectly bind to integrins and modulate the activation of the molecular pathway downstream to these receptors 8, 9. CD82, also known as KAI1, is a member of the tetraspanin superfamily and has been identified as a potent inhibitor of cancer metastasis. Consistently, downregulation of CD82 in different metastatic cancers as compared to primary tumors was reported 10, 11. Proteomics-based studies demonstrated that CD82 is glycosylated at three specific residues: Asn129, Asn157 and Asn198, which are located in the extracellular loop (ECL2) domain 12. However, the role of CD82 post-translational modification and its impact on ovarian cancer metastasis remain largely uncharacterized.

In this study, we demonstrate that glycosylation of CD82 at Asn157 is fundamental to ensure CD82-mediated inhibition of ovarian cancer cell migration and metastasis both *in vitro* and *in vivo*. Mechanistically, we show that CD82 glycosylation at Asn157 is required for efficient binding to integrin α5β1 and subsequent disruption of the integrin-based cell adhesion to fibronectin. We further uncovered a previously undescribed mechanism that MGAT3 is a critical glycosyltransferase, which promotes CD82 glycosylation in ovarian cancer cells. Interestingly, MGAT3 is significantly downregulated in ovarian cancer metastasis that contributes at least in part to provoke low glycosylation of CD82.

## Material and Methods

### Cell lines and culture

Human ovarian cancer cell lines ES2 and SKOV3 were purchased from ATCC and cultivated in RPMI-1640 medium supplemented with 10% fetal bovine serum (FBS) and 1% penicillin/streptomycin. HEK-293T cells were purchased from ATCC and maintained in Dulbecco's modified Eagle's medium (DMEM) supplemented with 10% FBS and 1% penicillin/streptomycin.

### Patient derived samples

Ovarian cancer clinical samples were obtained from The Central Maternity Hospital of Tianjin. This study was approved by the ethics committees of The Central Maternity Hospital of Tianjin. All patients signed an informed consent. Patients' information is reported in **Table S1**.

### Plasmids and transfections

The human CD82 cDNA was fused with Flag-peptide at the C-terminal and cloned into the lentiviral expression vector pLV-EF1α-MCS-IRES-Bsd/puro (Biosettia, San Diego, CA, USA). The CD82-Flag NQ mutants (N129Q, N157Q, N198Q, N157/198Q, 3NQ) were generated by site-directed mutagenesis using the pLV-CD82-Flag expression vector as a template. All constructs were verified by DNA sequencing. The primers used for the generation of the CD82 point mutants are listed in **Table S2.**

### Quantitative reverse transcription PCR (RT-qPCR)

Total RNA was extracted using TRIZOL reagent (Invitrogen) following manufacturer's instructions. cDNA was then synthesized using the Trans Script First-Strand cDNA Synthesis Super Mix Kit (Trans Gen Biotech, Beijing, China). qPCR was performed in a CFXTM real-time thermal cycler (Bio-Rad, Hercules, CA, USA) using a Trans Start Top Green qPCR Supper Mix kit (Trans Gen Biotech). Data analysis was performed with the comparative ΔCt method using GAPDH as internal control 13. The sequences of the primers used in this study are listed in **Table S3**.

### Generation of stable cell lines by lentiviral delivery system

Stable ovarian cancer cells expressing wild type CD82-Flag, the glycosylation deficient mutants CD82-Flag NQ or control cells were established using a lentiviral-based delivery system. For the generation of lentiviral particles, 4x10^6^ HEK293T cells were seeded in a 10 cm culture dish and cultivated overnight. Cells were co-transfected with 9 µg of empty or CD82-containing pLV-EF1α-MCS-IRES-Bsd/puro plasmids together with packaging and envelope vectors (4.5 µg pMDLg/pRRE, 1.8 µg pRSV-REV, 2.7 µg pCMV-VSV-G purchased from Addgene) using Lipofectamine 2000 (Invitrogen). Culture medium containing lentiviral particles was collected 48 h post-transfection and filtered through a 0.45 µm membrane. Lentiviral particles were diluted 1:2 in fresh medium and supplemented with 8 μg/mL polybrene. For lentiviral infection, target cells were incubated with lentiviral particles for 6-8 h. After incubation, cells were supplemented with fresh medium and incubated for additional 48 h. Stable cells were selected using puromycin (1 µg/mL).

### Animal experiments

BALB/c nude (nu/nu) mice were purchased from Vital River Laboratory Animal Technology Co. Ltd (Beijing, China) and maintained in pathogen-free facility of the Nankai University. All of the animal experiments were approved by the Nankai University Animal Care and Use Committee and handled according to the Nankai University Animal Welfare Guidelines.

For the mice xenograft model, 1×10^6^ ES2 cells stably expressing wild type (WT) CD82, the glycosylation deficient mutant N157Q or a control vector (MCS), were subcutaneously or intraperitoneally injected into 6-week-old female nude mice (n= 6 per group). Tumor volume was measured starting from day 10 post-injection every 5 days and calculated using the standard equation: V = 1/2 × L × W^2^, where V is the tumor volume, L the tumor length and W the tumor width. The number of metastatic foci were counted in three randomly selected fields and weighted.

### Western blot analysis and immunoprecipitation

Western blot analysis was performed as described previously 14. Briefly, adherent cells were washed twice with PBS and collected by scraping. Cellular pellets were re-suspended in RIPA lysis buffer (25 mM Tris-HCl pH 7.6, 150 mM NaCl, 1% sodium deoxycholate, 0.1% SDS) supplemented with protease inhibitor cocktail (Sigma‐Aldrich, St. Louis, MO, USA) and incubated on ice for 30 min. Protein lysates were cleared by centrifugation (16200 g for 15 min at 4 °C) and the protein concentration was assessed by BCA Protein Assay (Bio-Rad). Proteins were separated onto polyacrylamide gels and transferred to PVDF membranes. The protein samples were heated into 100 ℃ for 5 min and the blots were carried out in reducing condition. Membranes were blocked for 1h in 5% milk in TBS-T (w/v) and incubated overnight at 4 °C with primary antibodies on a shaking platform. On the next day, membranes were washed 5 times in TBS-T and incubated with appropriate HRP-conjugated secondary antibodies for 1 h. After incubation, membranes were extensively washed in TBS-T prior to signal detection using the Tanon Chemiluminescent Imaging System (Tanon, Shanghai, China). In these studies the following primary antibodies were used: Anti-Flag tag (Sigma-Aldrich; F1804), CD82 (CST; D7G6H), integrin β1 (SCBT; sc-8978), integrin α5 (CST; 4705S), p-FAK (CST; 3283S), FAK (CST; 3285S), p-SRC (CST; 2105S), SRC (CST; 2108S), paxillin (BD; 610620) integrin β4 (SCBT; sc-9090), Talin (SCBT; SC-15336), MGAT3 (proteintech; 17869-1-AP), Fibronectin (BD; 610078), Caveolin (SCBT; sc-984), Alix (SCBT; SC-99010), TSG101 (SCBT; SC-22774), p62 (SCBT; sc-166870), GM130 (SCBT; sc-55591).

For immunoprecipitation, cell lysates were collected in IP buffer (50 mM Tris-HCl; pH 7.4, 150 mM NaCl, 0.1% NP-40, 5 mM EDTA) supplemented with protease inhibitor cocktail (Sigma-Aldrich) and cleared by centrifugation. Protein lysates were incubated with specific antibodies as indicated in the figure legends or with non-immune immunoglobulin (IgG; negative control) and with G-agarose beads overnight on a rotating wheel. Next day, the beads were washed in ice-cold IP buffer, resuspended in 2x western blot loading buffer (65 mM Tris-HCl pH 6.8, 25% glycerol, 2% SDS, 0.01 % bromophenol blue, 50 mM DTT) and boiled at 95 °C for 5 min prior to western blot analysis.

### Transwell assay

Transwell assay was performed as already described with minor modifications 14. Briefly 2x10^5^ cells were resuspended in RMPI-1640 medium supplemented with 1% FBS and seeded in the upper chamber of a 24-well transwell plate with 8 μm pore size. The lower chamber was filled with RMPI-1640 supplemented with 10% FBS. 6 hours after seeding, the transmigrated cells were fixed with 4% paraformaldehyde and stained with crystal violet. Pictures were acquired using an Olympus BX51 microscope and the number of cells was counted using imaging J software.

### Cell adhesion and RGD blocking assay

96-well plates were coated with 10 mg/mL Fibronectin (FN, Millipore, Darmstadt, Germany) overnight at 4 °C. Ovarian cancer cell lines were stained with the fluorescent dye Calcein AM (abcam, MA, USA). For treatment with RGD-motif containing peptides (RGDs), Calcein stained cells were incubated with 1μg/mL RGDs (GL Biochem; Shangai) or BSA for 1 h at 37 ℃. After incubation, 5x10^3^ cells were seeded on fibronectin pre-coated plates for 1 h and unattached cells were removed by washing twice in PBS. Adherent cells were photographed using Olympus BX51 microscope and quantified by green fluorescence measurement using the Glomax 96 microplate luminometer (Promega, WI, USA).

### Live-cell imaging

Live-cell imaging for cell migration analysis was performed using AS MDW live-cell imaging system (Leica Microsystems) controlled by the Image Pro software (version 6.3; Media Cybernetics). Cells were cultured at 37 °C in a humidified 5% CO_2_ atmosphere. Bright-field images were acquired every 5 min over 4.5 h. Cell migration was tracked manually using the ImageJ plugin MTrackJ and plotted with the origin of migration superimposed at 0.0.

### High-content assay

High content assay was performed on 24-well plates pre-coated with 10 μg/mL fibronectin overnight at 4 °C. Cells were serum deprived for 8 h, washed twice in PBS, trypsinized with 0.25% trypsin and then re-suspended in RPM1-1640 medium supplemented with 1% FBS. 1x10^5^ cells were seeded in each well and the migration was monitored by incubation for 4 h at 37 °C by Operetta High-Content Imaging System (PerkinElmer, US) and analyzed by Harmony Analysis System (PerkinElmer, US).

### Immunofluorescence

Cells were seeded onto fibronectin pre-coated glass slides, fixed with 4% formaldehyde in PBS for 15 min and washed twice in ice-cold PBS. Cells were blocked for 1 h in 5% goat serum in PBS at room temperature for 1 h and incubated overnight at 4 °C in primary antibody. In this study, the following antibodies were used: Anti-Flag tag (Sigma-Aldrich; F1804), FAK (CST; 3285S), paxillin (BD; 610620). Next day, cells were washed 3 times in PBS and incubated with Alexa Fluor 488 or Alexa Fluor 594 conjugated antibodies (ZSGB-BIO, Beijing, China) for 1 h at room temperature. Cells were counterstained with DAPI. Pictures were captured using a confocal microscope (Olympus, Tokyo, Japan).

### Immunohistochemistry (IHC)

IHC staining was performed as described previously 14. Briefly, mice or human tissue sections were incubated with antibodies against CD82 or MGAT3 overnight at 4 °C. Next day, samples were incubated with a biotin-conjugated secondary antibody for 2 h at room temperature and then incubated with an avidin-biotin-peroxidase complex. The antibody detection was performed using 3-amino-9-ethylcarbazole chromogen.

### Hematoxylin and eosin (HE)

HE staining was performed using standard procedures. Briefly, the sections were deparaffinized by 2 times xylene, 5 min, and following 100% EtOH, 90% EtOH, 80% EtOH, 70% EtOH, 5 min, and 2 times H2O, 2 min each. Then, the sections were stained with hematoxylin-eosin kit (C0105, Beyotime, Shanghai, China). The sections were stained with hematoxylin solution for 1-2 min, and following the rinse with running tap water for 10 min. The sections were stained with eosin solution for 30 s to 2 min. Thereafter, the sections were dehydrated with 70% EtOH, 80% EtOH, 90% EtOH, 100% EtOH for 10 s, and 2 times xylene 5 min each. Finally, the sections were mounted with resinous mounting medium.

### Exosomes isolation, characterization and labelling

Exosomes were purified from cells cultured in serum-free medium for 36 h using already described procedures 15. Purified exosomes were suspended in physiological saline and characterized using limited trypsin digestion, immunoelectron microscopy (IEM), and western blotting. Limited trypsin digestion was performed as already described 16. Briefly, exosomes were incubated with 0.05% trypsin for 5 min at room temperature to digest surface proteins prior to western blot analysis.

IEM was performed using standard procedures. Purified exosomes were re-suspended in 2% paraformaldehyde and adsorbed into carbon-coated formvar EM grids (Electron Microscopy Sciences) for 20 min. The grids were then washed in physiological saline and transferred to 50 mM glycine/PBS for 3 min 3 times. The grids were washed in blocking buffer (5% BSA in PBS) for 10 min and incubated with 20 µl of anti-Flag antibody diluted in blocking buffer (1:200) for 30 min. The grids were transferred into 1% glutaraldehyde for 5 min and incubated with 20 µl of goat anti-mouse IgG/Gold antibody (Bioss, Beijing) for 30 min. Finally, the grids were embedded with 30μl of uranyl-oxalate solution for 90 s and air dried. Pictures were captured using FEI Talos™ F200C transmission electron microscope.

### Statistical analysis

Data are expressed as the average ± standard deviation of at least 3 independent experiments. Statistical significance was assessed using the Student´s t-test and GraphPad Prism 5 software (Inc., La Jolla, CA).

## Results

### CD82 is glycosylated in ovarian cancer cells

**Figure 1:**
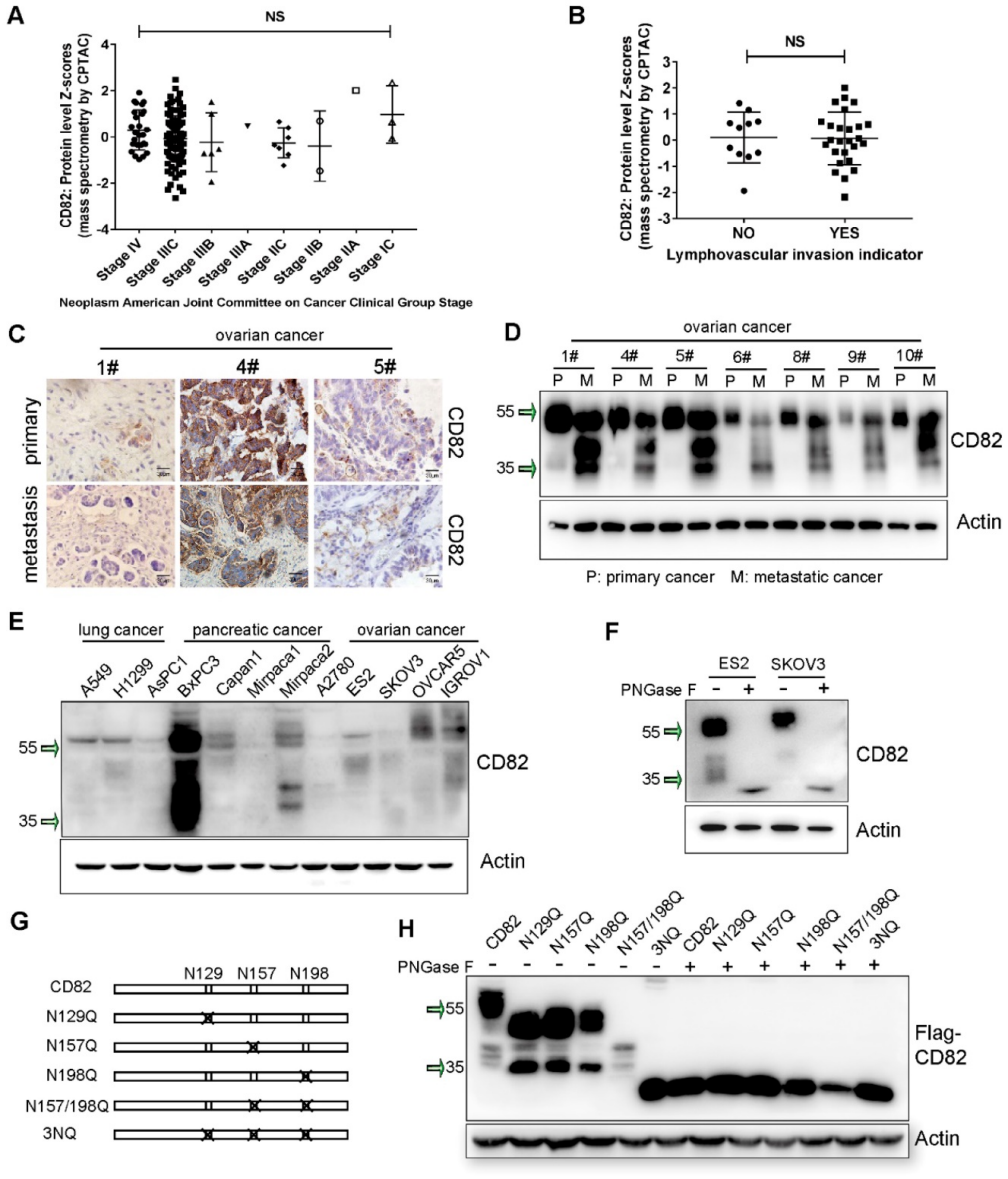
** CD82 is glycosylated in ovarian cancer. A.** Mass-spectrometry based proteomics data for CD82 expression in ovarian cancers at different progression stages was obtained from the Cancer Genome Atlas database (TCGA) and indicate no significant difference. Cancer staging was performed according to the American Joint Committee on Cancer (AJCC). NS: not significant.** B.** Mass spectrometry-based proteomics data indicate no significant difference in CD82 expression between ovarian cancers with or without lymphovascular invasion. **C.** Immunohistochemistry analysis of CD82 expression in clinical samples from primary and metastatic ovarian cancers. Note that CD82 expression is not significant change between primary tumors and metastasis. **D.** Western blot analysis of CD82 expression in primary and metastatic ovarian cancers. Note the reduction in the expression of the 55 kDa fraction of CD82 in metastasis and the concomitant increase in lower molecular weight fractions. **E.** Western blot analysis of CD82 expression in lung, pancreatic and ovarian cancer cell lines.** F.** ES2 and SKOV3 ovarian cancer cell lines lysates were either left untreated or incubated with recombinant glycosidase (peptide-N-glycosidase F; PNGase F) as indicated, prior to western blot analysis. A representative western blot of three independent experiments is shown. **G.** Schematic representation of the CD82 glycosylation deficient mutants used in this study. The crosses indicate substitutions of asparagine (N) with glutamine (Q). **H.** Cellular lysates derived from ES2 ovarian cancer cells stably expressing Flag-tagged CD82 and NQ point mutants were either untreated or incubated with PNGase F and analyzed by western blotting. Note the reduction in the molecular size of CD82 glycosylation deficient mutants as compared with CD82.

Numerous studies demonstrated that CD82 plays a pivotal role as inhibitor of tumor metastasis in various cancers 10, 17, 18. However, the role of CD82 in ovarian cancer metastasis has not been investigated. We explored CD82 expression using The Cancer Genome Atlas database (TCGA). No significant changes of CD82 expression were found at different stages of ovarian cancer (**Figure 1A**). Moreover, no association between CD82 expression and lymphovascular invasion was observed (**Figure 1B**). The expression of CD82 in human biopsies from primary and metastatic ovarian cancers was analyzed using IHC. We did not observe any significant change in CD82 expression in ovarian metastatic and paired primary tumors (**Figure 1C**, **S1A**). Interestingly, western blotting data indicate that CD82 mainly detected at around 55 kDa in primary tumors, while a reduction of 55 kDa and a significant increase of smaller between 35 and 55 kDa detected in metastatic tissues (**Figure 1D**, **S1B**). The mRNA levels of CD82 were no different in metastatic and primary tumors by real time PCR detection (**Figure S1C**).

Furthermore, expression pattern of CD82 in lung, pancreatic and ovarian cancer cell lines revealed a molecular weight ranging between 35 and 55 kDa (**Figure 1E**). It was previously demonstrated that tetraspanins are highly glycosylated proteins 9. We subjected protein lysates derived from ES2 and SKVO3 ovarian cancer cell lines to treatment with recombinant glycosidase (peptide-N-glycosidase F; PNGase F) to remove the presumed N-glycan structures. CD82 protein was detected at around 30 kDa in PNGase F treatment, while was detected at 55 kDa in untreated cells (**Figure 1F**).

Former proteomics based-analysis identified three major residues in the EC2 domain of CD82, which are N-glycosylated: Asn129, Asn157 and Asn198 (**Figure 1G**) 12. To prove whether the glycosylation is caused the separation pattern of CD82 in western blotting, we generated CD82 glycosylation deficient point mutants. Each asparagine (N) was substituted with glutamine (Q) to generate the single mutants N129Q, N157Q, N198Q, the double mutant N157/198Q and the triple mutant N129/N157/N198Q (3NQ) (**Figure 1G**). The five glycosylation deficient mutants and the CD82 were stably overexpressed in ES2 cells using a lentiviral-based system. The subsequent western blot analysis revealed that the abolishment of single or combined glycosylation residues generated protein bands at the lower molecular weight **(Figure 1H)**. The triple mutant 3NQ could be mainly detected at around 30 kDa. This molecular weight was also obtained with all glycosylation mutants upon treatment with PNGase F (**Figure 1H**). These results were confirmed by analysis of the glycosylation deficient mutants overexpressed in SKOV3 cells (**Figure S2A**). All these data corroborate that CD82 is highly glycosylated in ovarian cancer cells and indicate that the reduction of 55 kDa CD82 and the concomitant increase in smaller molecular weight fractions in metastatic ovarian cancer can be attributed to impaired CD82 glycosylation.

### Glycosylation of CD82 at Asn157 impairs ovarian cancer cells migration *in vitro*

**Figure 2:**
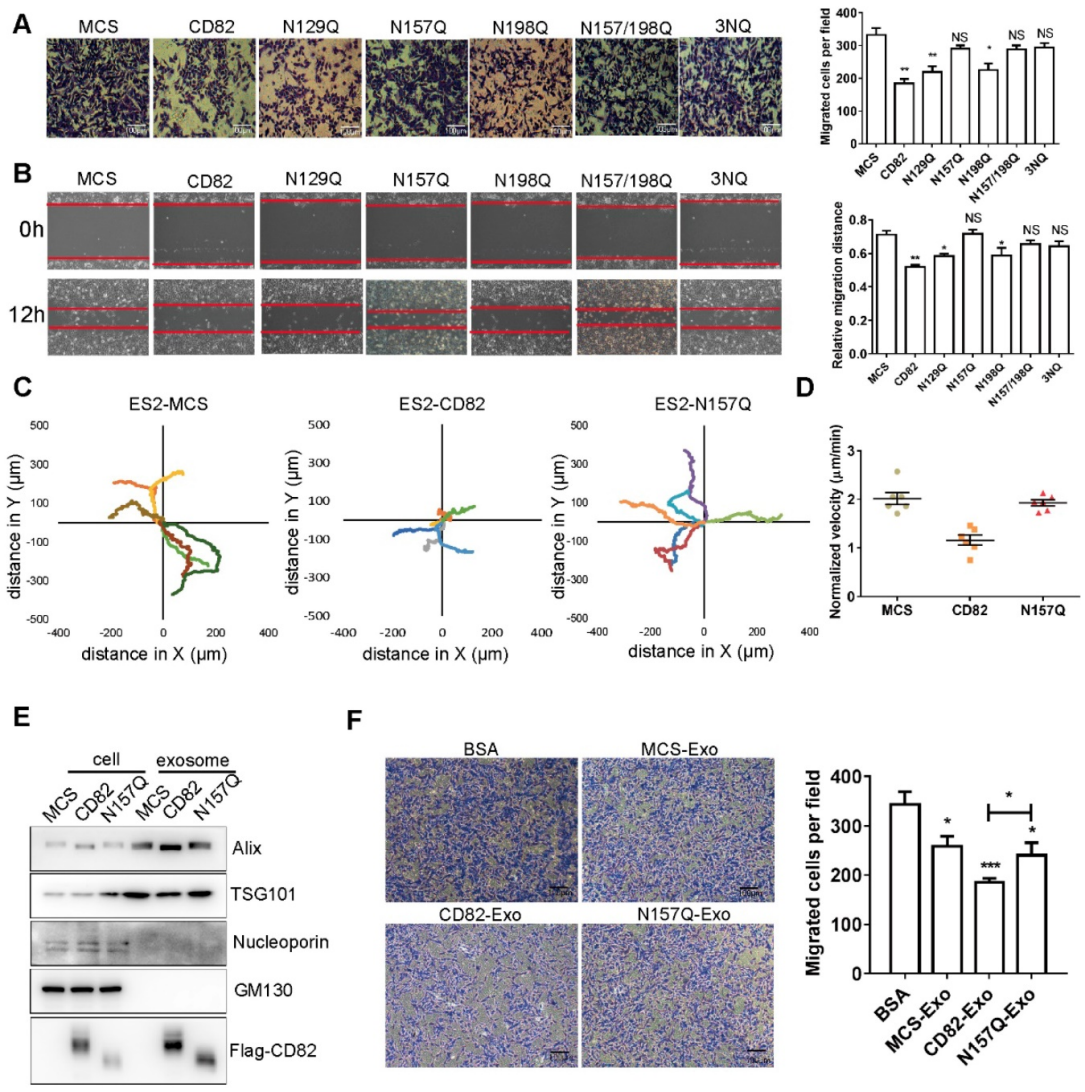
** Glycosylation at Asn 157 is required for CD82-mediated inhibition of ovarian cancer cell migration. A.** Transwell migration assay of stable control (MCS), CD82- and NQ mutants overexpressing ES2 cells. The transmigrated cells were stained with crystal violet (left panel). Scale bar 100 µm. A quantification of the number of migrated cells per field ± SD is shown in the histogram on the right. **p<0.01, *p<0.05, NS: not significant. **B.** Stable ES2 cells as in A were subjected to scratch assay. Representative pictures at time 0 and 12 h after scratching are shown. Red lines highlight the scratch margin. The relative distance of wound edges ± SD is given in the graph on the right (n=3). **p<0.01, *p<0.05, NS: not significant. **C.** Superimposed migration trajectories of stable control, CD82 and N157Q overexpressing ES2 cells as determined by live cell imaging. 6 cells for each group were analyzed (represented with different color lines). **D.** Normalized migration velocity of stable ES2 cells as in C. **E.** Western blot analysis of whole cell and exosomes protein lysates from stable ES2 cells. Western blot membranes where probed with antibodies against the exosomes markers Alix and TSG101 or against p62 and GM130 as negative control. CD82 overexpression was verified with anti-Flag antibody. **F.** Transmigration assay of ES2 cells incubated with control BSA or with exosomes derived from control, CD82 and N157Q overexpressing cells. A representative picture out of three independent experiments is shown on the left. A quantification of the average number of transmigrated cells per field ± SD of all performed experiments is shown in the histogram on the right. **p<0.01, *p<0.05.

We next investigated whether the glycosylation of CD82 has any impact on ovarian cancer cells migration. A transwell migration assay demonstrated that CD82, N129Q and N198Q mutants significantly reduced migration of ES2 cells. In contrast, the N157Q mutant, the double mutant N157/198Q and the triple mutant 3NQ did not exert any significant effect on migration of ES2 cells (**Figure 2A**). Similar results were observed in scratch assay **(Figure 2B**). Consistent results were also obtained in SKOV3 stable cell lines (**Figure S2D-E**). Taken together these data strongly imply that the glycosylation of CD82 at N157 is sufficient for CD82-mediated inhibition of ovarian cancer cells migration. To provide a more exact assessment of the migratory potential, we analyzed the migration distance of control, CD82 and N157Q mutant cells using time-lapse assay. As demonstrated in **Figure 2C** CD82 significantly reduced the migration of ES2 cells whereas no effect was caused by N157Q mutant. The cell migration videos and the relative quantification of the migration velocity (**Videos S1A-C**, **Figure 2D**) further confirmed these data.

Exosomes were isolated from control, CD82 and N157Q mutant cells. The characteristics were assessed using Alix and TSG101 as exosome markers and nucleoporin and GM130 as negative controls (**Figure 2E**, **S2B**). In addition, we confirmed the localization of CD82 in exosomes using immunoelectron microscopy (IEM) and limited trypsin digestion assay (**Figure S2C**, **S2G**). Next, we treated ES2 cells with exosomes, which were pre-labeled with Dir dye and confirmed the internalization of exogenous exosomal CD82 by immunofluorescence using anti-Flag antibody (**Figure S2H**). CD82-enriched exosomes significantly impaired ovarian cancer cell migration in contrast to exosomes derived from control and N157Q mutant cells (**Figure 2F, S2F**). Altogether these data demonstrate that CD82 glycosylation at N157 is a determinant post-translational modification responsible for CD82-mediated inhibition of ovarian cancer cell migration *in vitro*.

### CD82 glycosylation at Asn157 impairs fibronectin-integrin pathway

**Figure 3:**
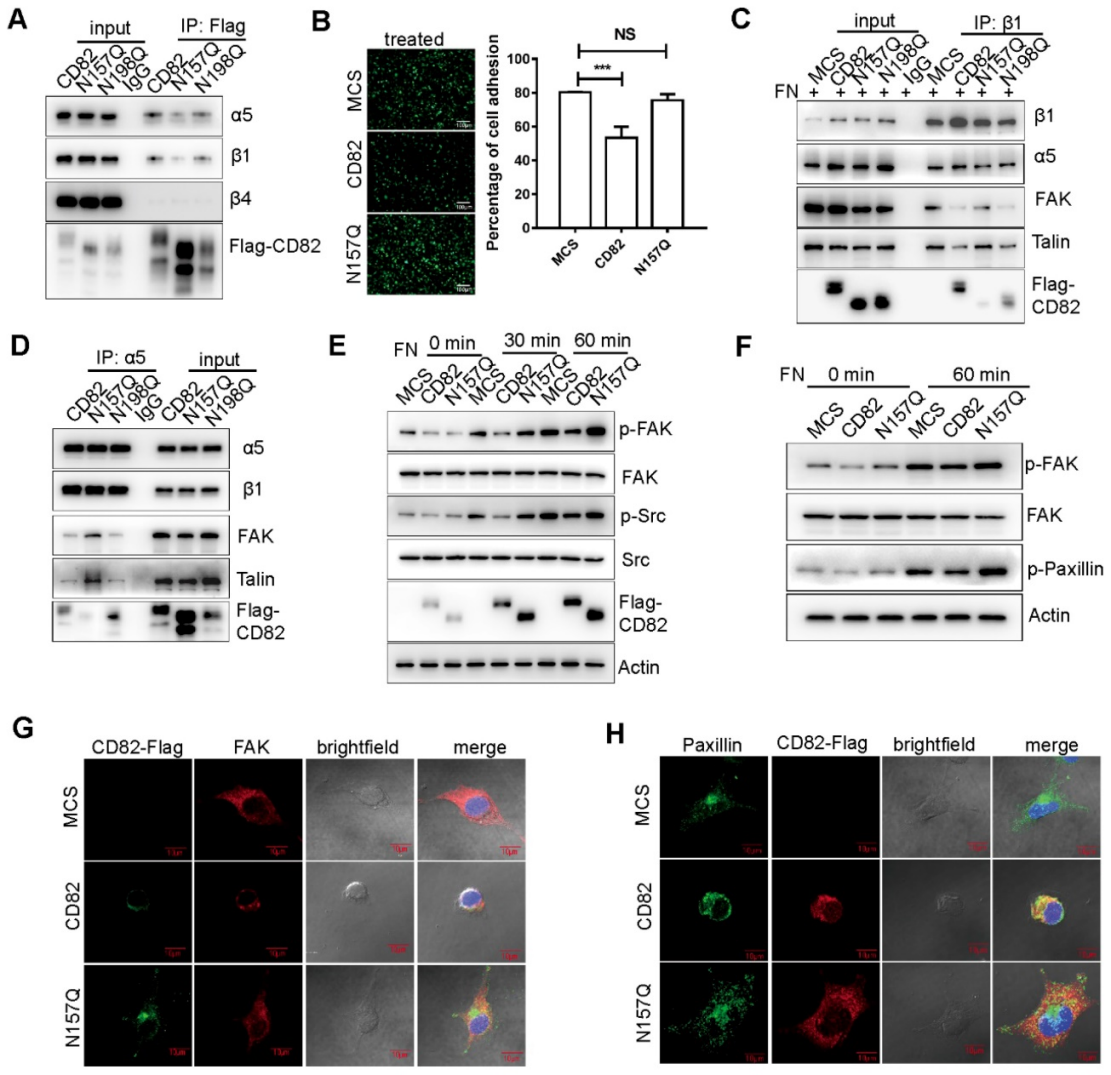
** CD82 glycosylation at N157 promotes CD82 binding to integrin α5β1 and disrupts the integrin-fibronectin signaling pathway. A.** Coupled immunoprecipitation (anti-Flag antibody; IP: Flag) and western blot analysis (anti-α5, β1 and β4 antibodies) of stable ES2 cells overexpressing Flag-tagged CD82, N157Q and N198Q mutants. **B.** Cell adhesion assay on fibronectin pre-coated plates that the stable control, CD82 or N157Q overexpressing cells were plated for 30 min. Adherent cells were visualized by Calcein fluorescence. A representative picture of three independent experiments is shown on the left. Scale bar 100 µm. Quantification of the relative fluorescence ± SD of three independent experiments is shown in the histogram on the right. ***p<0.005. NS: Not significant. **C.** Coupled immunoprecipitation (anti-β1 integrin antibody; IP: β1) and western blot analysis (anti-fibronectin; FN, α5, β1, FAK and Talin antibodies) of stable control (MCS) and CD82 overexpressing cells grown in presence of fibronectin. IgG was used as a negative control. A representative western blot out of three independent experiments is shown. **D.** Coupled immunoprecipitation (anti-α5 integrin antibody; IP: α5) and western blot analysis (β1, Paxillin, Talin, and anti-Flag antibodies) of cellular lysates derived from stable ES2 cells expressing CD82, N157Q and N198Q. A representative picture of three independent experiments is shown. **E.** Western blot analysis of phosphorylated and total FAK and Src in stable control and CD82 overexpressing cells at different time points after incubation with Fibronectin. A representative western blot of three independent experiments is shown. Note the reduction in the phosphorylation of FAK (p-FAK) and Src (p-Src) in CD82 as compared with MCS and N157Q expressing cells. **F.** Representative western blot (n=3) of phosphorylated and total FAK and phosphorylated paxillin (p-Paxillin) in stable ES2 cells upon treatment with fibronectin. Actin was used as a loading control. **G.** Representative immunofluorescence pictures of the subcellular localization of exogenous CD82 (Flag) and FAK in MCS, CD82 and N157Q overexpressing ES2 cells. Cellular nuclei were counterstained with DAPI. Scale bar 10 µm. **H.** Immunofluorescence analysis of Paxillin subcellular localization in MCS, CD82 and N157Q overexpressing cells (green). Exogenous CD82 was stained using anti-Flag antibody (red). Cell nuclei were counterstained with DAPI. Scale bar 10 µm. A representative picture of three independent experiments is shown.

We were further interested to get more insight into the molecular mechanism by which CD82 glycosylation at Asn157 inhibits ovarian cancer cells migration. Different studies demonstrated that tetraspanins function as scaffolding proteins for membrane receptors such as integrins, modulating thereby the activation of downstream signaling pathways 9. Integrins including α5β1, α4β1 and αvβ3 play pivotal roles in the anchorage of cells to the ECM especially through the binding to the extracellular matrix protein fibronectin 19-21. The binding of integrins to fibronectin is in turn essential for the transmission of intracellular signals, which promote cellular migration and motility 21. Since previous studies suggested that CD82 suppresses cell migration by association with integrins and disruption of the fibronectin-integrin axis 22, we reasoned that the glycosylation of CD82 at N157 might impact its interaction with integrins. Consistently with previous reports, we could show in co-immunoprecipitation experiments that Flag-tagged CD82 stably expressed in ES2 cells interacts with endogenous integrin α5β1 but does not interact with the β4 subunit (**Figure 3A**). Noticeably, we could further demonstrate that the glycosylation deficient mutant N157Q, but not the N198Q mutant, displayed an impaired capacity of binding to integrin α5β1, indicating that the glycosylation of CD82 at Asn157 is required for the efficient interaction of CD82 with integrins (**Figure 3A**).

Since integrin α5β1 contributes to cellular adhesion to fibronectin, we wondered whether the N157 glycosylated CD82 might inhibit the adhesion of ovarian cancer cells by disrupting the integrin-fibronectin interaction. Indeed, ES2 cells overexpressing CD82 possess an impaired capacity to adhere to fibronectin coated plates in contrast to control and N157Q overexpressing cells (**Figure 3B**,** S3A**). We could further confirm the inhibitory function of N157 glycosylation on cell adhesion using exosomes. Exosomes enriched in CD82 significantly inhibited ovarian cancer cells adhesion in contrast to control and N157Q enriched exosomes (**Figure S3B**). To univocally demonstrate that CD82 glycosylation at Asn157 is responsible for the disruption of the integrin-fibronectin interaction, we assessed this interaction in presence of CD82 and the N157Q and N198Q mutants. Since the interaction between integrins and fibronectin is pivotal for the recruitment of cytosolic integrin-binding adaptor proteins such as FAK and Talin 23, we also estimated the amount of FAK and Talin co-immunoprecipitated with integrin β1. A significantly reduced interaction of these molecules with integrin β1 in ES2 cells overexpressing CD82 and N198Q mutant but not in control and N157Q lent an additional support for the proposed inhibitory role of Asn157 glycosylation (**Figure 3C**). Consistently, co-immunoprecipitation experiments using anti-integrin α5 antibody demonstrated a dramatic increase in the association of integrin α5 with FAK and Talin in ES2 cells overexpressing the glycosylation deficient N157Q mutant as compared with CD82 and N198Q mutant overexpressing cells (**Figure 3D**).

Binding of integrins to fibronectin induces the recruitment and also phosphorylation of FAK, which in turn activates the integrin downstream pathway leading to the phosphorylation of adaptor proteins such as Src and Paxillin 23. To further confirm that the glycosylation of CD82 contributes to diminished activation of the integrin pathway, we analyzed the phosphorylation levels of FAK and Src in stably transfected ES2 cells upon treatment with fibronectin. As expected, cells overexpressing CD82 displayed a significant reduction in the phosphorylation of FAK and Src. While FAK and Src phosphorylation was clearly induced in response to fibronectin in control and N157Q mutant cells (**Figure 3E**). Consistently, a reduced phosphorylation of another down-stream integrin target, Paxillin, was observed in CD82 but not N157Q mutant overexpressing cells (**Figure 3F**). Since the interaction of integrin with fibronectin increases the recruitment of FAK and Paxillin to the focal adhesions, the subcellular localization of these molecules was estimated. In agreement with our expectations, FAK and Paxillin were mainly distributed at the cellular periphery in control and N157Q mutant expressing cells, while in CD82 overexpressing cells they were constricted rather to the perinuclear space (**Figure 3G-H**, **S3C**).

**Figure 4:**
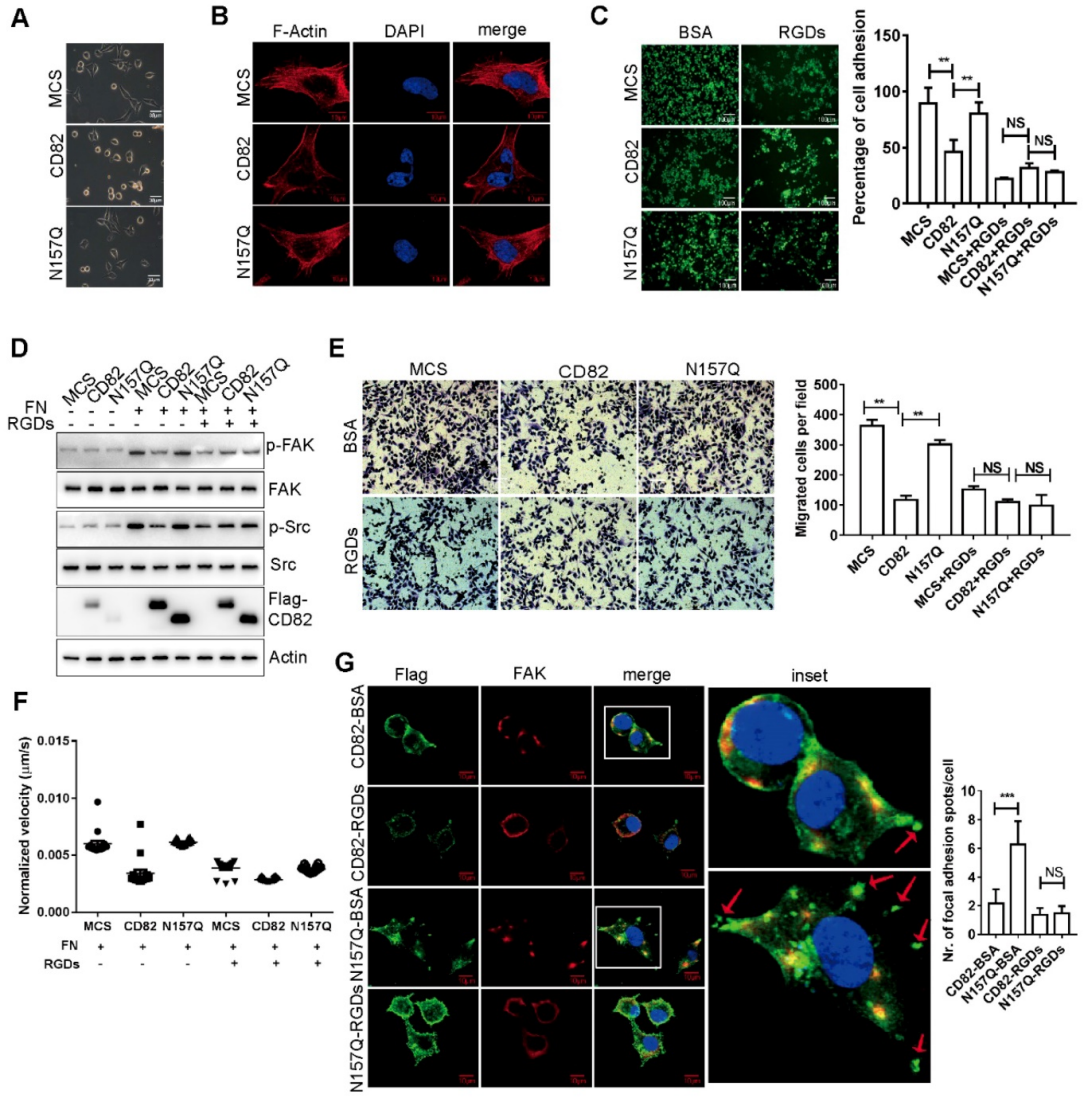
** RGD-loop containing peptides (RGDs) abolished the inhibition of CD82 on integrin signaling pathway. A.** Representative bright field image of cell spreading assay performed in stable ES2 cells. Note the inhibition of cell spreading in CD82 overexpressing cells as compared with MCS and N157Q. **B.** Representative fluorescent picture of stress fiber formation in stable ES2 cells as visualized by F actin staining. Cell nuclei were counterstained with DAPI. Scale bar 10 µm. **C.** Calcein stained ES2 cells were either pre-incubated with BSA or with RGDs and subjected to adhesion assay on fibronectin coated plates. A representative picture of three independent experiments is shown. A relative quantification of the number of adherent cells ± SD of three independent experiments is shown in the histogram on the right. **p<0.01, NS: not significant; scale bar 100 µm. **D.** Representative western blot analysis (n=3) of phosphorylated and total FAK and Src expression in stable ES2 cells upon treatment with fibronectin (FN) and RGDs. **E.** Representative pictures of transmigration assay performed in stable ES2 cells pre-incubated with BSA or RGDs. Quantification of the number of transmigrated cells ± SD is reported in the graph on the right. **p<0.01, NS: not significant**. F.** Representative graph of the relative migration velocity of stable ES2 cells in presence or absence of RGDs as established by high content analysis.** G.** Immunofluorescence analysis of FAK subcellular localization and the focal adhesion spots formation in stable ES2 cells overexpressing CD82 or N157Q after treatment with BSA or RGDs. Scale bar 10µm. Quantification of the number of focal adhesion spots per cell for 10 random fields is reported in the graph on the right. ***p<0.005, NS: not significant.

Moreover, since the generation of driving forces required for cellular migration results in cellular spreading, we evaluated the impact of CD82 glycosylation on the spreading capacity of ovarian cancer cell lines. In agreement with our assumption, CD82 overexpressing cells possess less spreading capacity as compared with control ES cells or N157Q mutant cells (**Figure 4A**). Because activation of integrin pathway has been connected to the formation of stress fibers, we further analyzed the F-actin organization. F-actin organization mainly produce and transmit mechanical tension required for cellular migration 24, 25. In fact, overexpression of CD82 but not N157Q mutant strongly inhibited stress fiber formation in ovarian cancer cells (**Figure 4B**,** S3D**).

Since it is known that fibronectin-derived Arg-Gly-Asp (RGD) motif-containing peptides can abolish the association of integrin α5β1 to fibronectin by competitive inhibition and therefore inhibit down-stream cellular events 26, 27, we analyzed the adhesion capacity of control, CD82 and N157Q mutant overexpressing cells on fibronectin coated plates upon treatment with RGD motif-containing peptides (RGDs) or in presence of BSA as a negative control. Noticeably, RGDs treatment strongly impaired cell adhesion in all cells tested (**Figure 4C**,** S3E**). However, while a reduced adhesion capacity was apparent in ES2 cells overexpressing CD82 upon treatment with BSA no further statistically significant differences were detected between the different cell lines after incubation with RGDs (**Figure 4C**, **S3E**). This finding strongly suggests that CD82 interrupts integrin-fibronectin contact efficiently enough to exclude the further interference by RGDs. Next, we estimated the extent of FAK and Src phosphorylation in stably transfected ES2 cells upon incubation with RGDs. In agreement with the previous data, cells overexpressing CD82 displayed a significant reduction in the activation of the integrin signaling pathway in absence of RGDs as compared to N157Q mutant and control cells. Treatment with RGDs peptides dramatically reduced the phosphorylation of FAK and Src in control and N157Q mutant cells but, again, no further reduction was detectable in CD82 overexpressing cells (**Figure 4D**). RGDs treatment and N157 glycosylation leads also to a slower rate of cell migration besides inhibiting cell adhesion. Indeed, using transwell and high-content analysis we could demonstrate that the RGD-mediated inhibition of the integrin-fibronectin binding in control and N157Q mutant overexpressing cells reduced the transmigration capacity and cellular migration velocity to the levels of untreated CD82 overexpressing cells and did not exert any additional inhibitory influence in these latter cells (**Figure 4E-F**,** S3F** and **Videos 2A-F**). Furthermore, the focal adhesion spots formation is a key step of cell adhesion or migration. We revealed that CD82 overexpressing cells significantly reduced the focal adhesion spots but not in N157Q mutant overexpressing cells (**Figure 4G**). The treatment with RGDs reduced the focal adhesion spots formation in both CD82 and N157Q mutant overexpressing cells (**Figure 4G**). All together these data strongly demonstrate that the glycosylation of CD82 at Asn157 inhibits cell motility and migration by disrupting the integrin-mediated anchorage to fibronectin leading thereby to a blunted activation of the signaling pathway downstream of integrin receptors.

### CD82 glycosylation at Asn157 inhibits ovarian cancer metastasis *in vivo*

**Figure 5:**
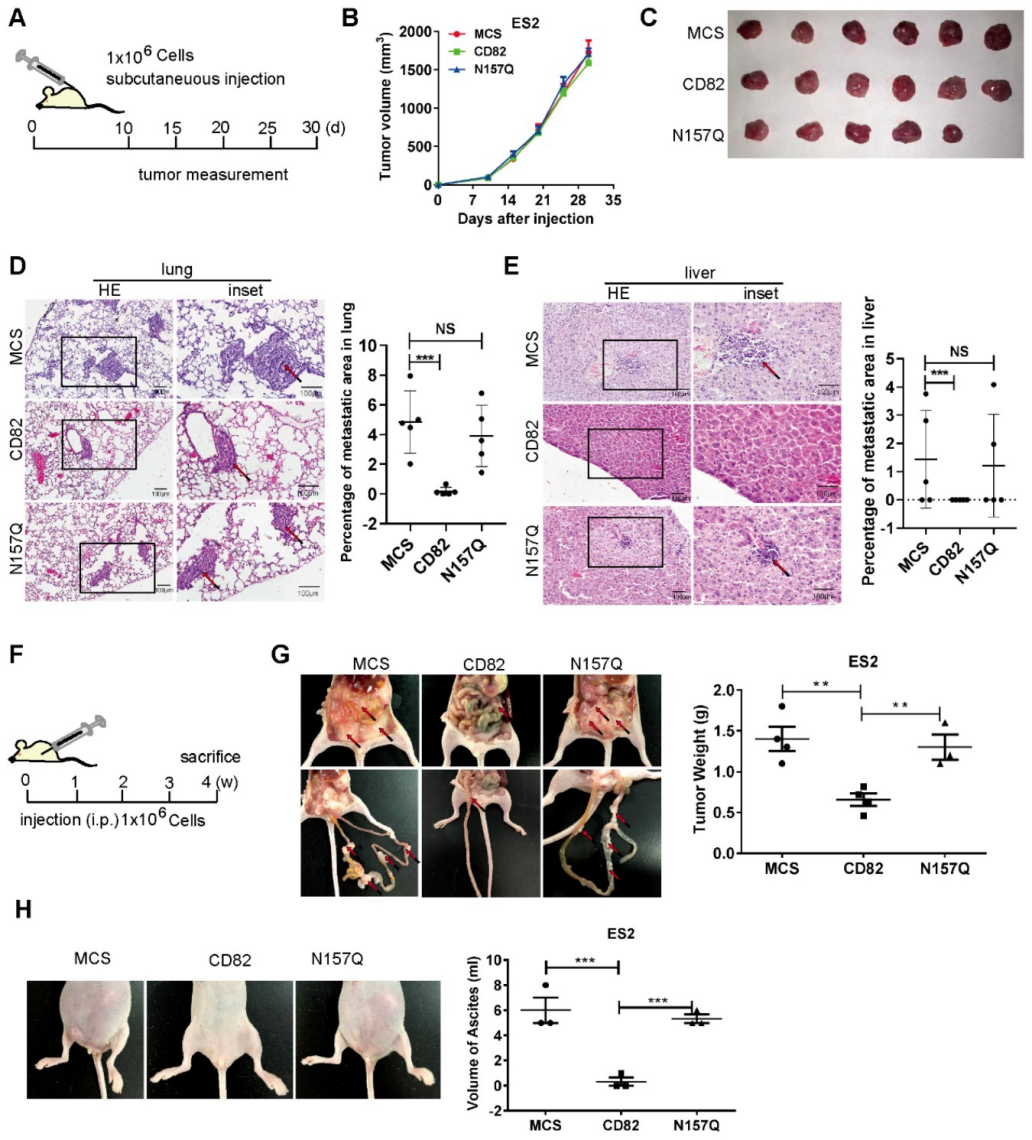
** Glycosylation of CD82 at N157 is fundamental for CD82-mediated inhibition of ovarian cancer metastasis *in vivo*. A.** Schematic representation of the subcutaneous mouse xenograft model. 1x10^6^ stable ES2 cells were injected subcutaneously in 4-6 weeks old female BALB/c nude mice. The tumor volume was measured every five days starting from day 10 post-injection. **B.** Graph representing the measurement of the tumor volume at the indicated time for the experiment described in a. **C.** Macroscopic images of excised tumors of the experiment described in a and b, 30 days after injection.** D-E.** Hematoxylin-eosin staining of lung and liver sections from the mouse xenograft model described in a. Note the impaired incidence of lung and liver metastasis in mice injected with CD82 overexpressing cells as compared with MCS or N157Q N=5. Quantification of the percentage of the metastasis area is reported in the graph on the right N=5. ***p<0.005, NS: not significant. **F.** Schematic representation of the intraperitoneal mouse xenograft model. 1x10^6^ stable ES2 cells were injected intraperitoneally in 4-6 weeks old female BALB/c nude mice. Mice were sacrificed 4 weeks post injection N=4.** G.** Macroscopic analysis of total tumor weight per mouse and mesenteric metastasis in the mouse xenograft model described in f. Quantification of the tumor weight per mouse is represented in the graph on the right. **p<0.01; N=4. **H.** Representative macroscopic pictures of ascites from mice xenografts described in f-g. Quantification of the total volume of ascites per mouse is shown in the graph on the right. N=3; ***p<0.005.

The results reported till now clearly demonstrate a pivotal role of CD82 glycosylation at N157 in the inhibition of ovarian cancer cell motility *in vitro*. We subcutaneously injected control, CD82 and N157Q mutant cells using a xenograft cancer model for 6 mice per group (N=6) in nude mice 28, 29 and analyzed the tumor growth (**Figure 5A**). According to the accepted role of CD82 as a metastasis suppressor, no impact on the growth rate of the primary tumors was discernible (**Figure 5B-C**). In agreement with the postulated CD82 function, mice injected with CD82 cells displayed a reduced incidence of lung and liver metastasis. Notably, mice injected with control or N157Q mutant cells showed higher metastasis formation (**Figure 5D-E**, **S4A** and **Table S4**). This finding provides evidence for the critical role of N157 glycosylation in metastasis inhibition *in vivo*.

To investigate the impact of glycosylated CD82 on the peritoneal dissemination of ovarian cancer, ES2 cells were injected intraperitoneally for 4 mice per group (N=4) and analyzed for tumor growth four weeks post-injection (**Figure 5F**). Mice injected with CD82 cells exhibited significantly reduced the tumor cell dissemination as compared with control or N157Q cells by the total tumor weight per mouse (**Figure 5G, S4B**). In addition, we demonstrate a clear reduction in the total size and volume of ascites per mouse injected with CD82 cells as compared with control or N157Q cells (**Figure 5H**). Our findings provide compelling evidence for a pivotal role of the glycosylation at N157 in CD82-mediated inhibition of ovarian cancer invasion and metastasis.

### MGAT3 mediates glycosylation of CD82 in metastatic ovarian cancer

**Figure 6:**
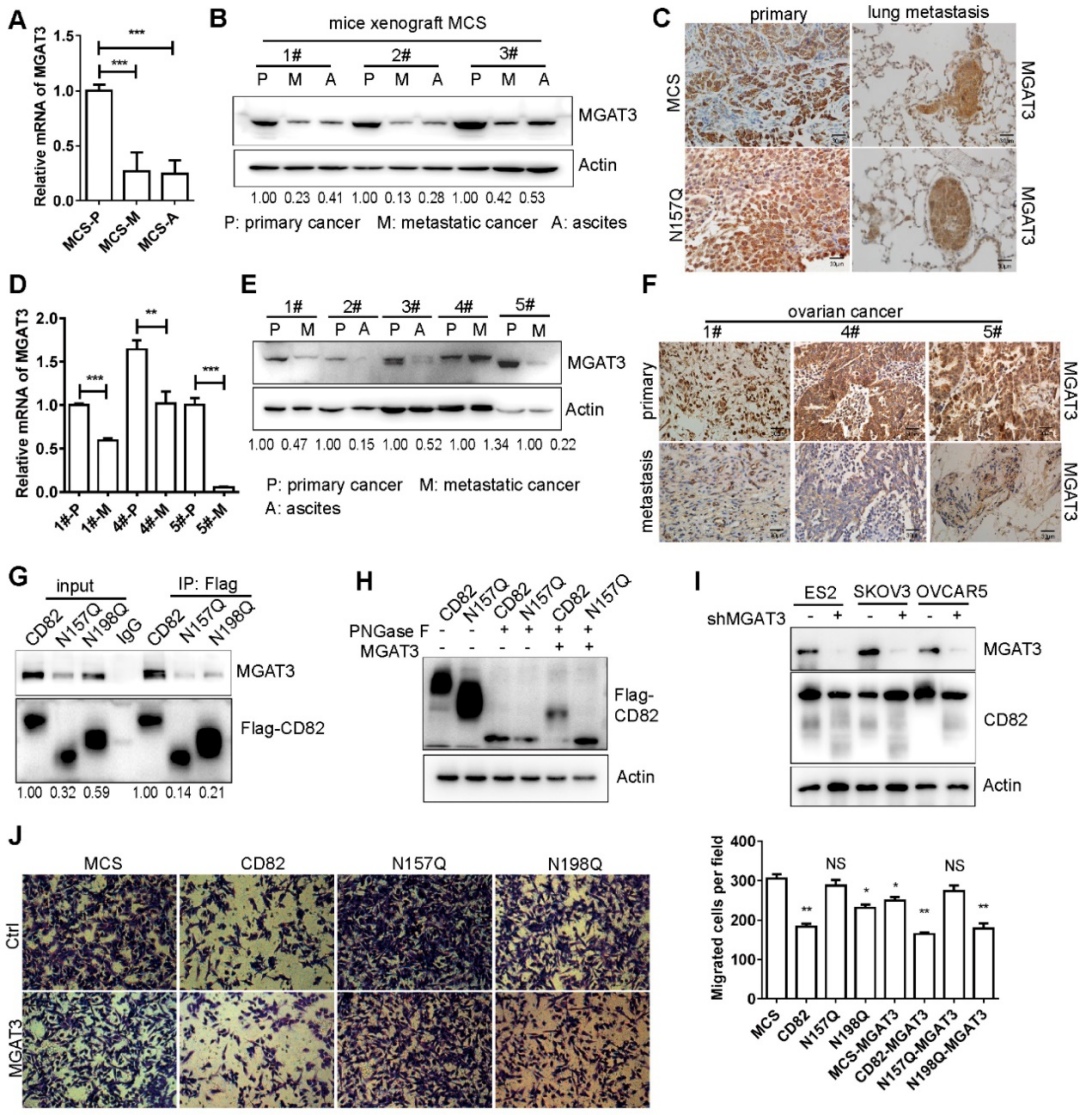
** MGAT3 impaired CD82 glycosylation and downregulation in metastatic ovarian cancers. A.** RT-qPCR analysis of MGAT3 expression in primary and metastatic tumors as well as in ascites derived from injection of MCS cells in the intraperitoneal xenograft model described in Figure. 4F-H. The relative expression of MGAT3 was calculated using the ΔCt method and GAPDH as normalization control. The graph represents the average of MGAT3 relative expression ± SD of 3 different mice. ***p<0.005. **B.** Western blot analysis of MGAT3 expression in primary (P) and metastatic (M) tumors as well as in ascites (A) of three different mice as in a. Actin was used as loading control. **C.** Immunohistochemistry analysis of MGAT3 expression in primary tumors and lung metastasis from MCS and N157Q mice in the intraperitoneal xenograft model described in Figure. 4F-H.** D**. RT-qPCR analysis of MGAT3 expression in clinical samples of primary (P) and metastatic (M) ovarian cancers. Expression of MGAT3 in three different patients (1#, 4# and 5#) is shown. The histogram represents the average of MGAT3 relative expression ± SD of three technical replicates for each patient. **p<0.01, ***p<0.005. **E.** Western blot analysis of MAGAT3 expression in primary and metastatic ovarian cancers as well as in ascites derived from different patients. **F.** Immunohistochemistry analysis of MGAT3 expression in primary ovarian cancers and lung metastasis of three different patients (1#, 4# and 5#). **G.** Coupled immunoprecipitation (anti-Flag antibody) and western blot analysis (anti-MGAT3 antibody) of cellular lysates derived from stable CD82, N157Q and N198Q overexpressing ES2 cells. The picture is representative of three independent experiments. **H.** Cellular lysates derived from stable ES2 cells overexpressing CD82 or N157Q were treated with recombinant MGAT3 or PNGase F as indicated prior to western blot analysis. **I.** MGAT3 knockdown was performed in three ovarian cancer cell lines: ES2, SKOV3, OVCAR5. The glycosylation of CD82 is impaired by the MGAT3 knockdown by western blot analysis. **J.** Transwell migration assay of MCS, CD82, N157Q and N198Q overexpressing ES2 cells transfected with an empty vector or with MGAT3 as indicated. The transmigrated cells were stained with crystal violet. A quantification of the number of migrated cells per field ± SD is reported in the histogram on the right. **p<0.01, *p<0.05, NS: not significant.

CD82 appears to be hypo-glycosylated in human metastatic ovarian tumors (**Figure 1D**). Thereafter we uncovered the possible cause responsible for CD82 hypo-glycosylation. Wang and collaborators have demonstrated that CD82 glycosylation mainly consists of N-acetylglucosamine, (α-2, 6) N-acetylneuraminic acid, and a core fucose 12. Noticeably, the modification is catalyzed by the enzyme MGAT3 30. Interestingly, studies reported an association between reduced expression of MGAT3 and cancer metastasis 31-36. A significant reduction of the mRNA and protein levels of MGAT3 was detected in metastatic tumors as well as in the ascites compared to primary tumors (**Figure 6A-B**, **S4C**). A lower MGAT3 expression was observed in lung metastasis compared with primary tumors using IHC (**Figure 6C**). In agreement with the results obtained in mice, a dramatic reduction of MGAT3 expression was detected in human metastatic ovarian tumors at the mRNA and protein levels (**Figure 6D-F**). A molecular complex formation between CD82 and MGAT3 would be required for CD82 glycosylation. Co-immunoprecipitation shows that endogenous MGAT3 efficiently co-precipitates with CD82 (**Figure 6G**, **S4D-E**). To unequivocally prove that MGAT3 is the enzyme responsible for CD82 glycosylation in ovarian cancer cells, we treated protein lysates from CD82 and N157Q mutant cells with recombinant PNGase F to remove the glycosylation moiety and subsequently subjected these lysates to treatment with recombinant MGAT3. In line with our expectation, treatment with recombinant MGAT3 after PNGase F addition increased the molecular weight of CD82 but not N157Q (**Figure 6H**). These data strongly indicate that MGAT3 specifically promotes CD82 glycosylation at N157. To verify the function of MGAT3 mediated CD82 glycosylation, we performed the MGAT3 knockdown and overexpression assays. Knockdown MGAT3 in the ovarian cancer cell lines using shRNA impaired the glycosylation of CD82 (**Figure 6I**). The overexpression of MGAT3 significantly reduced the transmigration capacity of control, CD82, and N198Q mutant but not of N157Q mutant cells (**Figure 6J**). The efficient overexpression of MGAT3 was confirmed by western blotting (**Figure S4F**). These data clearly demonstrate that MGAT3 is responsible for CD82 glycosylation and suggest that MGAT3 downregulation in metastatic ovarian cancer may promote metastasis.

## Discussion

CD82 is a ubiquitously expressed transmembrane protein which has been implicated in a vast number of biological functions such as immune-response, differentiation, cancer growth and metastasis 9. Numerous reports demonstrated that CD82 is a potent inhibitor of cancer metastasis and downregulated in metastatic cancers 10, 11, 37. The mechanisms that lead to CD82 downregulation in cancer still remain poorly characterized. Promoter hypermethylation, gene mutations and loss of heterozygosity only infrequently provoke CD82 downregulation in cancers, although reduced CD82 promoter activity due to aberrant availability of specific transcription factors might account for this process 38-42. In addition, impaired CD82 protein stability might contribute to CD82 downregulation in some metastatic tumors 43.

CD82 is a highly post-translational modified protein which is mainly N-glycosylated and palmitoylated 10. The essential role of glycosylation for CD82-mediated functions was proposed by Ono and collaborators. The authors demonstrate that the effect of CD82 in the inhibition of cellular motility requires the restoration of efficient N-glycosylation 44. Marjon et al. recently demonstrated that N-glycosylation and palmitoylation of CD82 are critical modifications that control CD82-dependent regulation of bone marrow homing of acute myeloid leukemia (AML). The authors demonstrated that glycosylation and palmitoylation exert opposite functions in the trafficking of AML cells in the bone marrow through differential regulation of membrane clustering of the adhesion molecule N-cadherin 45. Zhou et al. demonstrated that the palmitoylation of CD82 is required for its inhibitory role on migration and invasion of prostate cancer cell lines 46. The subcellular distribution of CD82 and its association with other tetraspanins are dependent on the palmitoylation. Palmitoylation regulates the specific tetraspanin-enriched microdomains formation or disrupts other tetraspanin/tetraspanin interactions 47. CD82 inhibits cancer cells dissemination from primary tumors by promoting cell-cell adhesion 48. Numerous studies demonstrated that CD82 plays a pivotal role as inhibitor of cancer cells motility by interfering integrin-mediated cellular adhesion and migration 49-53. CD82 promotes integrin α6 internalization resulting thereby in reduced adhesion of cancer cells to laminin, and subsequent reduction of cell migration 54. In addition, CD82 inhibits cell surface expression of β1 integrin by reducing its glycosylation and maturation in prostate cancer cell lines 55, 56. Moreover, studies demonstrated that CD82 impacts the expression and activation of adaptor proteins downstream of the integrin pathway 56, 57. It is also known that CD82 regulates the localization in microdomains and the ubiquitylation of EGFR. EGFR also impact CD82 regulated integrin signaling to impair the cell adhesive/invasive ability 58.

In this study, we dissected a previously undescribed mechanism controlling ovarian cancer metastasis. We demonstrated that glycosylation of CD82 at Asn 157 is a fundamental post-translational modification which is required for CD82-mediated inhibition of ovarian cancer metastasis *in vitro* and *in vivo*. These findings are in line with previous data obtained in ldlD cells that the extensive N-glycosylation impairs CD82 interaction with α3 and α5 integrins 59. Numerous glycoproteins are recognized as possible markers for cancer diagnosis and prognosis 60-63. Studies reported that the glycosyltransferase MGAT3 controls cancer proliferation, migration and metastasis although these effects may be beneficial or detrimental in a cell type-dependent manner 64-70. Another glycosyltransferase MGAT5 has also been reported to catalyze β1-6 GlcNAc-branched N-glycans, which is increased in highly metastatic tumor cell lines 71, 72. MGAT3 transfers GlcNAc to a β1-4 mannose in N-glycans to form a bisecting GlcNAc, which may suppress β1-6 GlcNAc branching formation catalyzed by MGAT5. We find that the glycosylation of CD82 is mainly catalyzed by the glycosyltransferase MGAT3. Interestingly, we additionally demonstrated that treatment of ovarian cells with CD82-enriched exosomes efficiently inhibits ovarian cancer cells adhesion and migration *in vitro* that may represent novel strategies for ovarian cancer metastasis therapies.

## Abbreviations

ECM: Extracellular matrix
EC1/2: Extracellular loop 1/2
FAK: Focal adhesion kinase
FBS: Fetal bovine serum
DMEM: Dulbecco's modified Eagle's medium
WT: Wild type
IP: Immunoprecipitation
IF: Immunofluorescence
IHC: Immunohistochemistry
H&E: Hematoxylin and eosin
IEM: Immunoelectron microscopy
TCGA: The Cancer Genome Atlas database
RGD: Arg-Gly-Asp
