# Supplementary material for: MGAT3-mediated glycosylation of tetraspanin CD82 at asparagine 157 suppresses ovarian cancer metastasis by inhibiting the integrin signaling pathway
Source: Theranostics. 2020 May 16;10(14):6467–82. doi: 10.7150/thno.43865 (PMC7255015; doi:10.7150/thno.43865)
Supplement: Supplementary file 1 — Supplementary figures and tables. [file thnov10p6467s1.pdf]

Supplementary Data

Supplementary tables

Table S1. Information of ovarian cancer clinical samples

Patient No.	Age	Gender	Diagnosis	Clinical samples
1	62	Female	IIIC	Primary/Metastatic cancer
2	51	Female	IIIB	Primary cancer/Ascites
3	52	Female	IIIC	Primary cancer/Ascites
4	66	Female	IIIC	Primary/Metastatic cancer
5	59	Female	IIIB	Primary/Metastatic cancer
6	68	Female	IIIC	Primary/Metastatic cancer
7	52	Female	IIIB	Primary cancer/Ascites
8	61	Female	IIIB	Primary/Metastatic cancer
9	50	Female	IIIC	Primary/Metastatic cancer
10	61	Female	IIIB	Primary/Metastatic cancer
11	52	Female	IIIC	Primary cancer/Ascites
12	61	Female	IIIB	Primary cancer
13	52	Female	IIIC	Primary cancer
14	61	Female	IIIB	Primary cancer
15	62	Female	IIIB	Primary cancer
16	46	Female	IIIC	Primary cancer
17	49	Female	IIIB	Primary cancer

Table S2. Primers for CD82 cloning and generation of CD82 point mutants

Gene	Primer	Sequences (5′-3′)
CD82	F	GCTCTAGAGCCACCATGGGCTCAGCCTGTATCAAAG
	R	CGACGCGTTCACTTATCGTCGTCATCCTTGTAATCGTACTTGGG GACCTTGCTG
N129Q	F	CGAGACTACCAAAGCAGTCGC
	R	GCGACTGCTTTGGTAGGTCTCG
N157Q	F	CAGCTTCTACCAATGGACAGAC
	R	GTCTGTCCATTGGTAGAAGCTG
N198Q	F	GCCCCCGGCCAAAGGACCCAG
	R	CTGGGTCCTTTGGCCGGGGGC

Table S3. Primers used for RT-qPCR analysis

Gene	Primer	Sequences (5'-3')
CD82	F	TGGACATCATTCGCAACTACAC
	R	GCATGGGTAAGTGGTCTTGGTA
MGAT3	F	GGACTTACTCCCTTTTGTGCA
	R	GTGGCCCAGTTCTAATCCGT
GAPDH	F	CTCTGATTTGGTCGTATTGGG
	R	TGGAAGATGGTGATGGGATT

Table S4. Incidence of lung and liver metastasis upon subcutaneous injection of stable control, CD82 and N157Q overexpressing ES2 cells

	**Incidence**		
**Site**	MCS	CD82	N157Q
Lung	100% (6/6)	17% (1/6)	80% (4/5)
Liver	100% (6/6)	17% (1/6)	80% (4/5)

Supplementary figure and figure legends

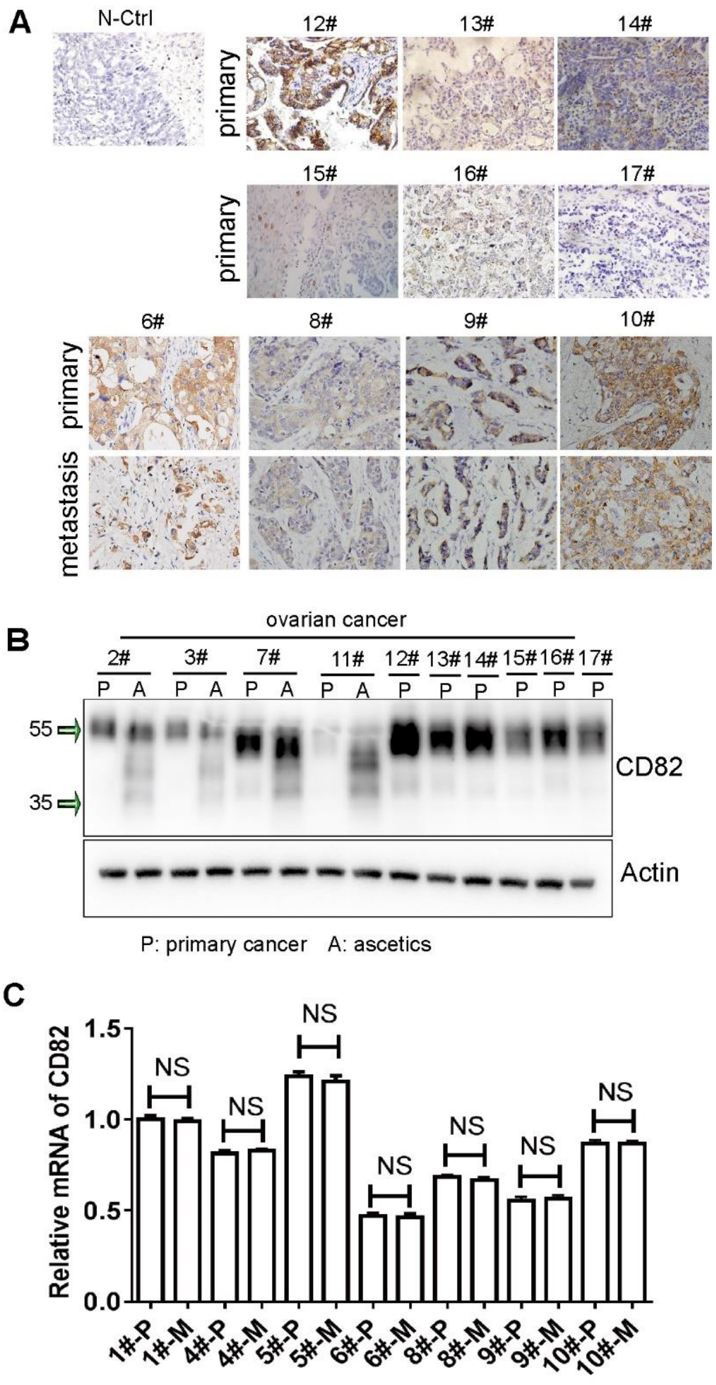

Figure. S1. **The protein status of CD82 in the samples of ovarian cancer patients**

A. Immunohistochemistry analysis of CD82 expression in clinical oavrian cancer samples. Negative control shows the section without primary anti-CD82 antibody incubation. Note that CD82 expression is diversity in the primary tumor from different patients. The expression of CD82 is not significant change between the paired primary and metastatic sample in the same patient. **B.** Western blot analysis of CD82 expression in primary and ascetics of ovarian cancers. Note the 55 kDa fraction of CD82 is enriched in primary tumor while the the reduction fraction of CD82 is increased in ascetics samples. **C.** The relative mRNA level of CD82 is measured by

qPCR in the paired primary and metastatic sample in the same patient. Note that CD82 expression is not significant change between the primary and metastatic cancer.

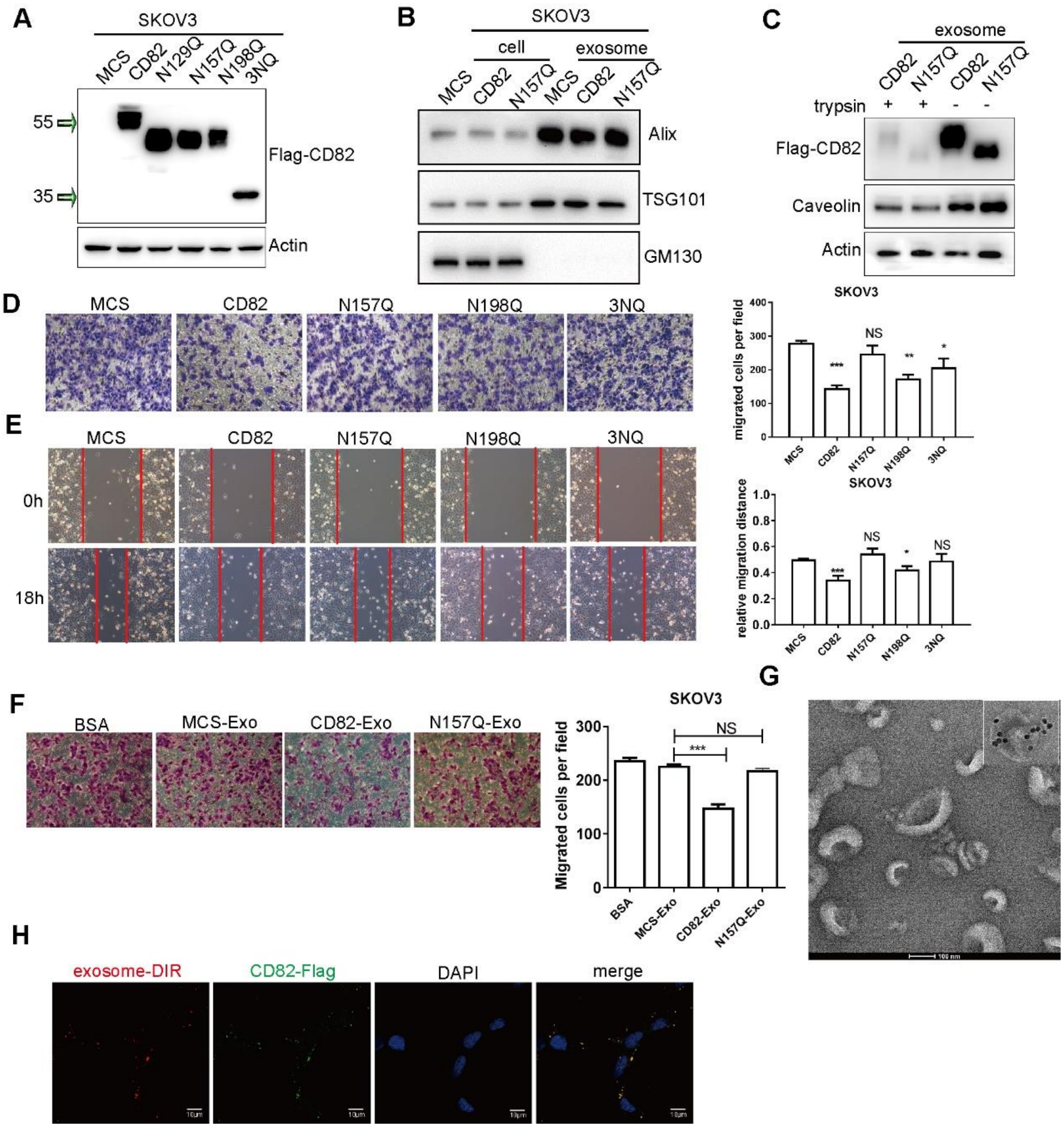

Figure. S2. **CD82 glycosylation at Asn157 inhibits SKOV3 ovarian cancer cells migration.**

A. Western blot analysis of Flag-tagged CD82 in SKOV3 stable ovarian cancer cell lines expressing MCS, CD82, and NQ glycosylation deficient mutants. **B.** Western blot analysis of whole cell and exosomes protein lysates from stable SKOV3 cells. Western blot membranes where probed with antibodies against the exosomes markers Alix and TSG101 or against GM130 as negative control. **C.** Representative western blot analysis of exosomes isolated from stable ES2 cells overexpressing CD82 and N157Q. Exosomes were subjected to limited trypsin digestion or left untreated, as indicated, prior to western blot analysis. Exogenous Flag-CD82 was detected using anti-Flag antibody. Note that trypsin efficiently digested the surface associated protein caveolin as well as exogenous CD82 but did not digest actin, which is confined to the

inner compartment of exosomes. **D.** Transwell migration assay of MCS, CD82 and NQ glycosylation deficient mutants overexpressing SKOV3 ovarian cancer cells. The transmigrated cells were stained with crystal violet (left panel). Scale bar 100 µm. Quantification of the number of migrated cells per field ± SD is reported in the histogram on the right. *** $p<0.005$, **$p<0.01$, *$p<0.05$, NS: not significant. **E.** Stable SKOV3 cells as in D were subjected to scratch assay. Representative pictures at time 0 h and 12 h hours after scratching are shown. Red lines highlight the scratch margin. The relative distance of wound edges ± SD is represented in the graph on the right (n=3). ***$p<0.005$, *$p<0.05$, NS: not significant. **F.** Transmigration assay of SKOV3 cells incubated with control BSA or with exosomes derived from control, CD82 and N157Q overexpressing cells. A representative picture out of three independent experiments is shown on the left. A quantification of the average number of transmigrated cells per field ± SD of all performed experiments is shown in the histogram on the right. ***$p<0.005$, NS: not significant. **G.** Representative immunoelectron microscopy image of purified exosomes derived from stable ES2 cells overexpressing Flag-tagged CD82. Exogenous Flag-CD82 was stained using anti-Flag antibody. Note the enrichment of CD82 on the surface of exosomes. Scale bar 100 nm. **H.** Exosomes isolated from stable CD82 overexpressing cells were pre-stained with DIR dye (red). ES2 cells lines were incubated for 3 h with pre-stained exosomes. After incubation cells were fixed and subjected to immunofluorescence analysis using anti-Flag antibody to detect internalized exogenous and exosome-derived CD82. A representative immunofluorescence picture is shown. Cell nuclei were counterstained with DAPI. Scale bar 10 µm.

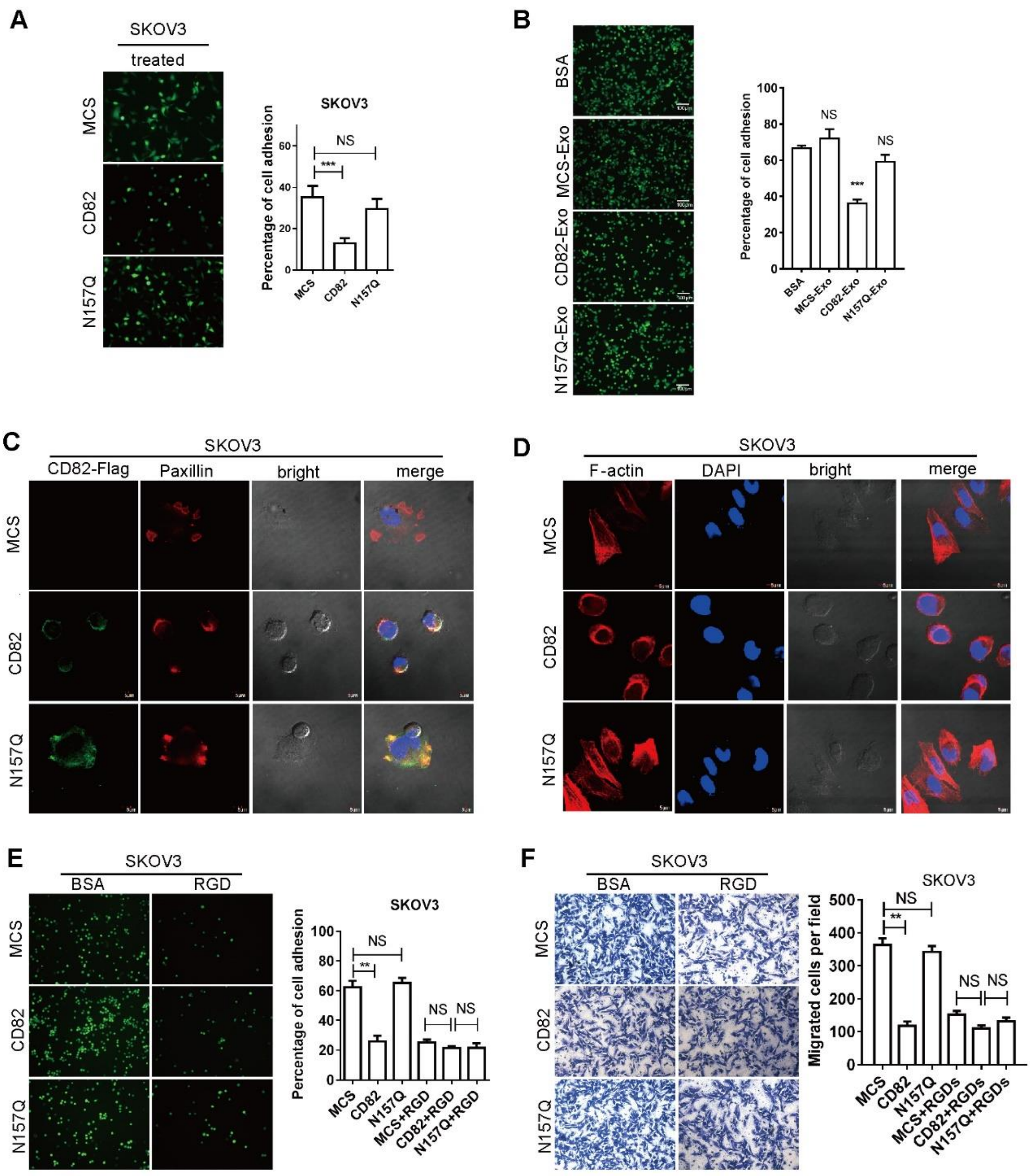

Figure. S3. Glycosylation of CD82 at Asn 157 inhibits activation of the integrin signaling pathway in SKOV3 cell lines.

A. SKOV3 cell adhesion assay on fibronectin pre-coated plates that the MCS, CD82 or N157Q overexpressing cells were plated for 30 min. Adherent cells were visualized by Calcein fluorescence. A representative picture of three independent experiments is shown on the left. Quantification of the relative fluorescence ± SD of three independent experiments is shown in the histogram on the right. ***$p<0.005$. NS: Not significant. **B.** Analysis of ES2 cells adhesion capacity on fibronectin coated plates upon treatment with BSA or with exosomes derived from MCS, CD82 and N157Q overexpressing cells. ES2 cells were incubated for 3 h with purified exosomes and stained with calcein. After incubation, cells were seeded on fibronectin coated plates and adherent cells were visualized and photographed using a fluorescent microscope.

A representative picture of three independent experiments is shown on the left. Quantification of the percentage of adherent cells ± SD of all performed experiments is shown in the histogram on the right. ***p<0.05; NS: not significant. **C.** Immunofluorescence analysis of Paxillin subcellular localization in MCS, CD82 and N157Q overexpressing cells (red). Exogenous CD82 was stained using anti-Flag antibody (green). Cell nuclei were counterstained with DAPI. Scale bar 5 μm. A representative picture of three independent experiments is shown. **D.** Representative fluorescent picture of stress fiber formation in stable SKOV3 cells as visualized by F actin staining. Cell nuclei were counterstained with DAPI. Scale bar 5 μm. **E.** Calcein stained SKOV3 cells were either pre-incubated with BSA or with RGDs and subjected to adhesion assay on fibronectin coated plates. A representative picture of three independent experiments is shown. A relative quantification of the number of adherent cells ± SD of three independent experiments is shown in the histogram on the right. **p<0.01, NS: not significant. **F.** Representative pictures of transmigration assay performed in stable SKOV3 cells pre-incubated with BSA or RGDs. Quantification of the number of transmigrated cells ± SD is reported in the graph on the right. **p<0.01, NS: not significant**.**

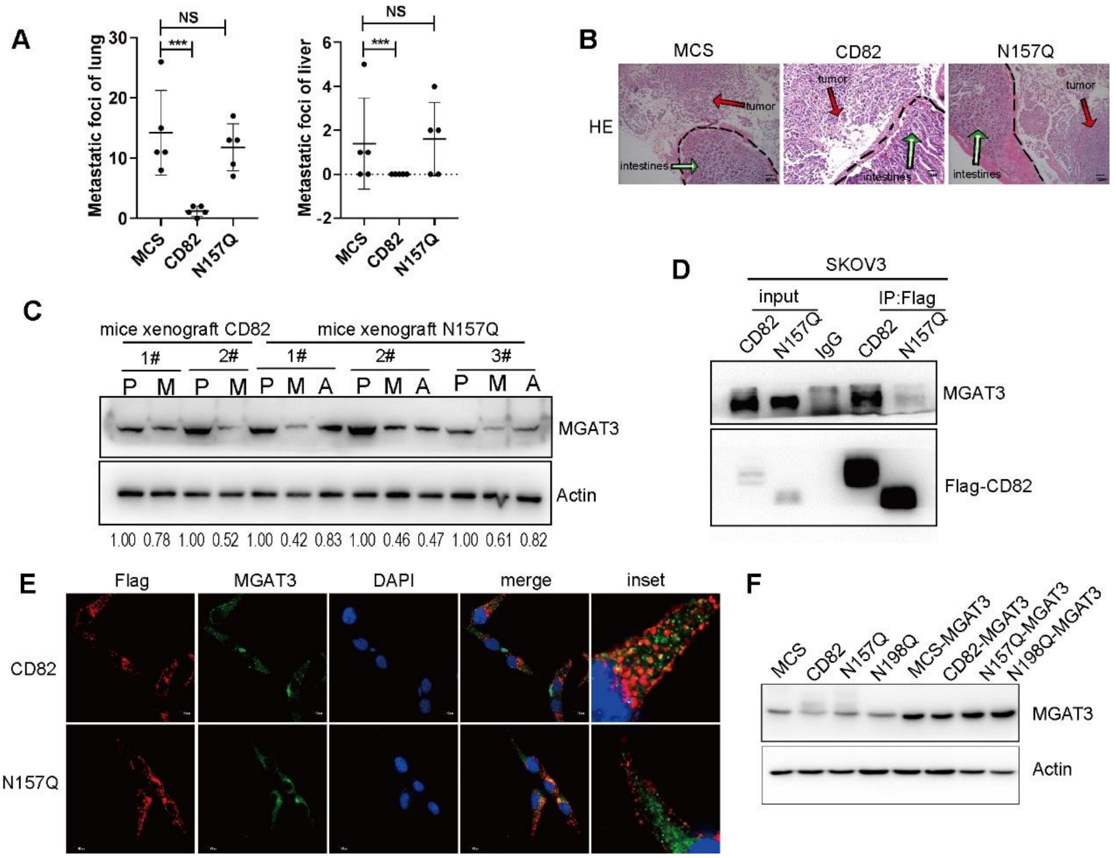

Figure. S4. MGAT3 regulated CD82 glycosylation to mediate ovarian cancer metastasis

A. The metastatic foci in lung and liver is measured in the subcutaneous mouse xenograft model. Quantification of metastatic foci number is reported in the graph. ***p<0.005, NS: not significant. Note the impaired incidence of lung and liver

metastasis in mice injected with CD82 overexpressing cells as compared with MCS or N157Q. **B.** Hematoxylin-eosin staining of the intestines with metastatic ovarian caner in intraperitoneal mouse xenograft model. **C.** Western blot analysis of MGAT3 expression in primary (P) and metastatic (M) tumors as well as in ascites (A) of the different mice beared with CD82 or N157Q overexpressing ES2 cells in the intraperitoneal xenograft model. Actin was used as loading control. **D.** Coupled immunoprecipitation (anti-Flag antibody) and western blot analysis (anti-MGAT3 antibody) of cellular lysates derived from stable CD82, N157Q and N198Q overexpressing SKOV3 cells. **E.** Immunofluorescence analysis of MGAT3 subcellular localization in MCS, CD82 and N157Q overexpressing cells (green). Exogenous CD82 was stained using anti-Flag antibody (red). Cell nuclei were counterstained with DAPI. Note CD82 is partly colocalizated with MGAT3 in cytoplasm and reduced in N157Q. **F.** Western blot analysis of cellular lysates derived from stable ES2 cells transfected with an empty vector or MGAT3 overexpressing plasmid.

Supplementary videos

Video. S1A

Timelapse video of the migration of stable control (MCS) ES2 cells.

Video. S1B

Timelapse video of the migration of stable CD82 overexpressing ES2 cells.

Video. S1C

Timelapse video of the migration of stable N157Q overexpressing ES2

cells.

Video. S2A

High-Content Analysis of the migration of stable control (MCS) ES2 cells on fibronectin coated plates.

Video. S2B

High-Content Analysis of the migration of CD82 overexpressing ES2 cells on fibronectin coated plates.

Video. S2C

High-Content Analysis of N157Q overexpressing ES2 cells on fibronectin coated plates.

Video. S2D

High-Content Analysis of the migration of stable control (MCS) ES2 cells on

fibronectin coated plates in precence of RGD-loop containing peptides.

Video. S2E

High-Content Analysis of the migration of CD82 overexpressing ES2 cells on fibronectin coated plates in precence of RGD-loop containing peptides.

Video. S2F

High-Content Analysis of N157Q overexpressing ES2 cells on fibronectin coated plates in precence of RGD-loop containing peptides.
